# Poisson Plus Quantification for Digital PCR Systems

**DOI:** 10.1038/s41598-017-09183-4

**Published:** 2017-08-29

**Authors:** Nivedita Majumdar, Swapnonil Banerjee, Michael Pallas, Thomas Wessel, Patricia Hegerich

**Affiliations:** ThermoFisher Scientific, Life Sciences Group, South San Francisco, 94080 USA

## Abstract

Digital PCR, a state-of-the-art nucleic acid quantification technique, works by spreading the target material across a large number of partitions. The average number of molecules per partition is estimated using Poisson statistics, and then converted into concentration by dividing by partition volume. In this standard approach, identical partition sizing is assumed. Violations of this assumption result in underestimation of target quantity, when using Poisson modeling, especially at higher concentrations. The Poisson-Plus Model accommodates for this underestimation, if statistics of the volume variation are well characterized. The volume variation was measured on the chip array based QuantStudio 3D Digital PCR System using the ROX fluorescence level as a proxy for effective load volume per through-hole. Monte Carlo simulations demonstrate the efficacy of the proposed correction. Empirical measurement of model parameters characterizing the effective load volume on QuantStudio 3D Digital PCR chips is presented. The model was used to analyze digital PCR experiments and showed improved accuracy in quantification. At the higher concentrations, the modeling must take effective fill volume variation into account to produce accurate estimates. The extent of the difference from the standard to the new modeling is positively correlated to the extent of fill volume variation in the effective load of your reactions.

## Introduction

Digital PCR is a state-of-the-art technique for measuring nucleic acid concentrations with high precision and accuracy. The digital method distributes target molecules into a large number of partitions such that each partition gets a number of molecules (0, 1, 2, etc.), theoretically following a Poisson distribution. Performing PCR on these partitions results in amplification being detected (positives) in those partitions containing one or more target molecules and no amplification being detected (negatives) in those partitions containing zero molecules. As positives may contain more than one copy of the target molecule, a simple summing of the number of positives will not yield the correct number of target molecules present across the partitions. Poisson statistics are widely employed to estimate the total number of target molecules present within the interrogated sample. For a detailed review of standard digital PCR modeling and characteristics, refer to ref. 1. In digital PCR data analysis, the number of molecules per unit partition is estimated from the fraction of partitions not recording a molecule over an ensemble of partitions. The estimate is then divided by partition volume to obtain a measure of the concentration. In this case, for the purposes of computation, an ensemble of identically sized partitions is assumed where the occurrence of a molecule in each partition follows a Poisson process. But this technique is adversely affected, particularly at higher concentration, when partitions are not identically sized.

**Figure 1:**
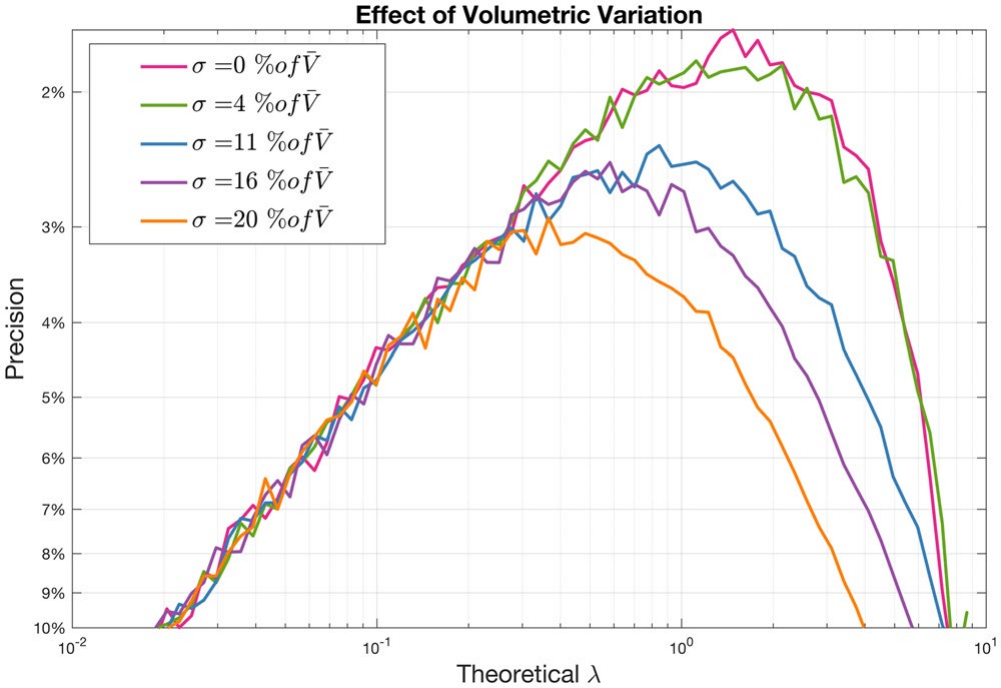
Effect of Partition Size Variation on Precision: Partition Size Variation affects precision significantly at higher concentrations^1^.

The effect of partition size variation is investigated with Monte Carlo simulations. Figure 1 shows the measurement precision for each λ (molecules per partition) computed using 20000 partitions. In this simulation, the concentration of the target molecules is kept constant. The average number of molecules in a partition is proportional to the volume of the partition. Precision is defined as the absolute value of the maximum deviation from the true rate (measured across the inner 90th percentile of simulation results) divided by the true rate. (Lower computed values, rising up the y-axis, are associated with higher precision and better measurement reproducibility). A normal distribution of partition sizes is assumed with the standard deviation taken as a percentage of the mean partition size.

Partition size variation impacts results for higher concentrations more significantly than for lower concentrations. The extent of the effect is positively correlated with the magnitude of partition size variation. This paper demonstrates how Poisson modeling can be extended to better estimate concentration when effective loads are not identically distributed. Current platforms supporting digital PCR use either array-based or droplet-based mechanisms to divide input sample into a large number of partitions. While array based systems have the advantage of fixed partition sizes, other factors can influence the effective load. If the effective load variation can be well characterized, adoption of the model proposed in this paper will enable precise measurements of target molecules despite load volume dissimilarities.

## Results

### The Poisson-Plus Model

#### The Poisson-Plus Approximation Model

For the standard Poisson model, the mean number of molecules per partition (*λ*) is assumed to be constant. For the new model, it is assumed that the *λ* in each partition is proportional to the size of the partition volume *v* as given as follows:

1 $${\rm{\lambda }}(v)=\mathrm{Cv}\,$$

The constant of proportionality *C* in (1) is the average number of molecules per unit volume, or, the concentration. The joint probability distribution of a partition to both not contain a molecule (indicated by the phrase ‘neg’) and be of size *v* is constructed using Bayes’ theorem^2^, given as follows:

2 $${\rm{P}}({\rm{neg}},{\rm{v}})={\rm{P}}(\mathrm{neg}|v){\rm{P}}({\rm{v}})$$

where *P*(*neg*|*v*) is the probability of a partition to register zero molecules for a given volume of the partition, and *P*(*v*) is a suitable probability density function for the partition volumes. *P*(*neg*|*v*) is the standard Poisson distribution with *λ* given by (1), evaluated at number of molecules = 0:

3 $${\rm{P}}({\rm{neg}}|{\rm{v}})={{{\rm{e}}}^{-{\rm{Cv}}}\frac{{({\rm{Cv}})}^{{\rm{n}}}}{{\rm{n}}!}|}_{{\rm{n}}=0}={{\rm{e}}}^{-{\rm{Cv}}}$$

For *P*(*v*), a normal distribution is first investigated. A normal distribution for variable *v* is a reasonable choice since nature abounds in examples in which quantities of interest are normally distributed. *P*(*v*), under assumptions of being normally distributed, is given by (4), where v_0_ is the mean and σ is the standard deviation:

4 $${\rm{P}}({\rm{v}})=\frac{1}{{\rm{\sigma }}\sqrt{2{\rm{\pi }}}}{{\rm{e}}}^{-\frac{-{({\rm{v}}-{{\rm{v}}}_{0})}^{2}}{2{{\rm{\sigma }}}^{2}}}$$

Note that the variable *v* in (4) can take negative values, which is unphysical so far as the problem at hand is concerned, since volume cannot be negative (addressed in the Poisson-Plus Model). Therefore, (4) is at best an approximation of the actual probability distribution followed by the partition volumes.

Substituting (3) and (4) into (2),

5 $${\rm{P}}({\rm{neg}},\,v)={{\rm{e}}}^{-{\rm{Cv}}}{{\rm{e}}}^{-\frac{-{({\rm{v}}-{{\rm{v}}}_{0})}^{2}}{2{{\rm{\sigma }}}^{2}}}\frac{1}{{\rm{\sigma }}\sqrt{2{\rm{\pi }}}}$$

A theoretical expression for *P*(*neg*) is obtained by integrating the volume variable out from the above expression, yielding:

6 $${\rm{P}}({\rm{neg}})={\int }_{-\infty }^{\infty }{\rm{P}}({\rm{neg}},{\rm{v}}){\rm{dv}}={{\rm{e}}}^{(\frac{1}{2}{{\rm{\sigma }}}^{2}{{\rm{C}}}^{2}-{{\rm{Cv}}}_{0})}$$

For an experimentally measured value of *P*(*neg*), *C* can be computed using (6). The closed form expression for C, as calculated from (6), is given as follows:

7 $${\rm{C}}=\frac{{{\rm{v}}}_{0}\pm \sqrt{{{\rm{v}}}_{0}^{2}+2{{\rm{\sigma }}}^{2}\,\mathrm{ln}\,{\rm{P}}({\rm{neg}})}}{{{\rm{\sigma }}}^{2}}$$

It can be shown that approximating the effective partition volumes by a normal distribution is not a serious deficiency in our model as long as the spread or the standard deviation of the partition sizes is small, or more precisely, the ratio of the standard deviation to the mean, $\frac{\sigma }{{v}_{0}}$, is sufficiently small. By sufficiently small, we mean enough that the left tail of the normal distribution goes close to zero for positive values of volume. A theoretical limit is imposed by $P(neg)=\exp (-\,0.5\ast {(1/\frac{\sigma }{{v}_{0}})}^{2})$ as the term inside the square root in (7) starts to go negative beyond this point, yielding physically meaningless imaginary results.

Of these two solutions for C, only one is valid and the other is unphysical. To determine which solution should be retained, the σ → 0 limit of this expression is investigated. In that limit, C should be independent of σ as per the first order approximation and should resemble the expression obtained from using the Poisson distribution with no partition size variability. In (7) the solution for C with the negative sign reduces to the expected limit as σ goes to zero. It can be shown that for σ → 0, C with the negative sign in (7) becomes independent of σ and reduces to the same form, as in the case of standard Poisson formalism, given below.

8 $${\rm{C}}=\frac{-\,\mathrm{ln}\,{\rm{P}}({\rm{neg}})}{{{\rm{v}}}_{0}}$$

Hence the realistic solution for *C* is given by

9 $${\rm{C}}=\frac{{{\rm{v}}}_{0}-\sqrt{{{\rm{v}}}_{0}^{2}+2{{\rm{\sigma }}}^{2}\,\mathrm{ln}\,{\rm{P}}({\rm{neg}})}}{{{\rm{\sigma }}}^{2}}$$

This solution is referred as “The Poisson Plus Approximation Model”, a complete derivation of which is provided in Additional Information, Appendix A. The term “approximation” is used to indicate that the mathematics used for the derivation of C has not taken care of the issue of negative partition sizes.

#### The Poisson-Plus Model

In the more rigorous “Poisson Plus” model, it is assumed that the partition size follows a truncated normal distribution $\hat{\aleph }(v)$ with the parameter *v*_0_ and *σ*. A truncated Normal distribution is given as follows:

10 $$\hat{\aleph }({\rm{v}})=\frac{1}{{\rm{\sigma }}\sqrt{\frac{{\rm{\pi }}}{2}}{\rm{erfc}}(-\frac{{{\rm{v}}}_{0}}{\sqrt{2}{\rm{\sigma }}})}{{\rm{e}}}^{-\frac{-{({\rm{v}}-{{\rm{v}}}_{0})}^{2}}{2{{\rm{\sigma }}}^{2}}}$$

The variable *v* in the above expression does not take negative values. Being a probability distribution function, the distribution satisfies the normalization condition ${\int }_{0}^{\infty }\hat{\aleph }({v}_{0},\sigma )dv=1$. Note that, *v*_0_ and *σ* are the parameters of the distribution, which, although related to the mean and the standard deviation of the measurements of partition size, are not exactly the same as those quantities.

Using (3) for *P*(*neg*|*v*) and (10) for *P*(*v*) in (2), the joint probability density of a partition not containing any molecules and being of size *v* is given as follows:

11 $${\rm{P}}({\rm{neg}},{v})=\frac{1}{{\rm{\sigma }}\sqrt{\frac{{\rm{\pi }}}{2}}{\rm{erfc}}(-\frac{{{\rm{v}}}_{0}}{\sqrt{2}{\rm{\sigma }}})}{{\rm{e}}}^{-{\rm{Cv}}}{{\rm{e}}}^{-\frac{-{({\rm{v}}-{{\rm{v}}}_{0})}^{2}}{2{{\rm{\sigma }}}^{2}}}$$

Next, the variable *v* can be integrated out as in (6) to yield the probability of negatives only:

12 $${\rm{P}}({\rm{neg}})={\int }_{0}^{\infty }{\rm{P}}({\rm{neg}},{\rm{v}}){\rm{dv}}$$

Using (11) in (12) and carrying out the integration (as shown in Additional Information, Appendix B ^3^), the final expression for *P*(*neg*) is given as follows:

13 $${\rm{P}}({\rm{neg}})=\frac{{\rm{erfc}}[-\frac{1}{\sqrt{2}}(\frac{{{\rm{v}}}_{0}}{{\rm{\sigma }}}-C{\rm{\sigma }})]}{{\rm{erfc}}[-\frac{1}{\sqrt{2}}(\frac{{{\rm{v}}}_{0}}{{\rm{\sigma }}})]}{e}^{-{{\rm{Cv}}}_{0}+\frac{1}{2}{{\rm{\sigma }}}^{2}{{\rm{C}}}^{2}}$$

For a given *P*(*neg*), *C*, the quantity of interest, has to be solved for implicitly by using numerical methods. This solution is referred as “The Poisson Plus Model”. This version is implemented in QuantStudio 3D AnalysisSuite^TM^ Cloud Software from Thermo Fisher Scientific^4, 5^, where this algorithm was first released to the public in July 2015. In the software, the mean and standard deviation from empirical measurements (discussed in the next section) are directly used for v_0_ and σ as given in above equations. For most practical purposes this is adequate. Simulation investigations indicate that as long as the standard deviation to the mean ratio is under some reasonable limit, the parameters *v*_0_ and *σ* closely follow the mean and the standard deviation of the truncated Gaussian distribution. In the unlikely event of further variation, one needs to specifically estimate v_0_ and *σ*, calculated from the mean and standard deviation of available data, for use in the model.

#### The Poisson Plus Approximation as a Limiting Case of the Poisson Plus Model

It is noted here that as $\frac{\sigma }{{v}_{0}}\,\to 0$, the multiplicative factor $\frac{erfc[-\frac{1}{\sqrt{2}}(\frac{{v}_{0}}{\sigma }-C\sigma )]}{erfc[-\frac{1}{\sqrt{2}}(\frac{{v}_{0}}{\sigma })]}$, appearing in (13), goes to 1. Hence (13) reduces to (6). This verifies that, as expected, “Poisson Plus Approximation” emerges as an approximation of the “Poisson Plus” model.

An alternate model for accommodating partition size variation was proposed by Huggett, *et al*.^6^, heretofore referenced as the HCF (Huggett-Cowen-Foy Model). The next section presents a comparison of performance across the two versions of the Poisson Plus, the HCF and the Poisson models.

#### Simulation Results

Monte Carlo simulations were run to demonstrate the remedial effects of quantification using alternate modeling that accounts for non-uniform partition size in Poisson processes. These simulations assume that the probability of a partition of capturing the molecule is proportional to the size of the partition. A normal distribution of partition sizes is assumed with the standard deviation taken as a percentage of the mean partition size. The steps of generating the simulation data are outlined in Additional Information, Appendix C.

**Figure 2:**
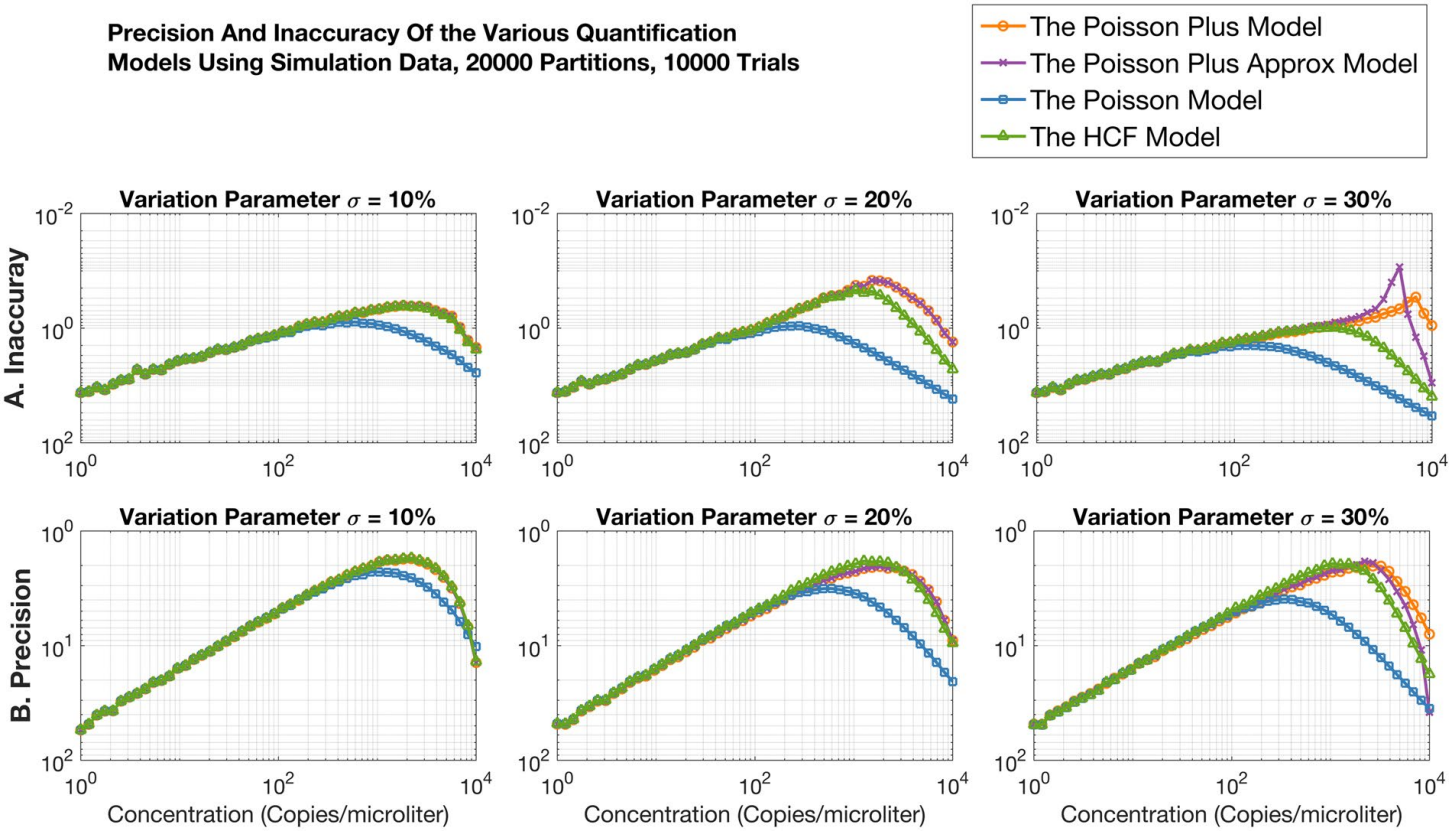
Comparison of performance across the two versions of the Poisson Plus, the HCF and the Poisson models in terms of A. Inaccuracy and B. Precision. The lower these values the better. All models taking partition size variation into account perform better than standard Poisson when variation in partition size is present, beyond 400 copies/microliter. The Poisson Plus models have better accuracy beyond 1000 copies/microliter for more than 20% variation.

Results from the Poisson Plus, the Poisson Plus Approximation, the Poisson and the HCF models are compared in Fig. 2 in terms of the measurement inaccuracy and precision. Inaccuracy is measured as the ratio of the absolute difference of the mean quantification from the trials and the expected concentration, to the expected concentration. The lower the inaccuracy the better. Precision is measured as the ratio of the absolute difference of the 95% confidence bound of the quantification across the trials and the expected concentration, to the expected concentration. The lower the precision the better.

Three levels of variation are considered: 10%, 20% and 30% of the mean partition size. Note that typical fill volume variation in QuantStudio 3D chips is under 10%. The higher levels of fill volume variation used in these simulations demonstrate the capabilities of the model. An N of 20000 partitions was used in the simulation. Each simulation was run for 10000 iterations. All models taking partition size variation into account perform better than standard Poisson when variation in partition size is present, beyond 400 copies/microliter. The Poisson Plus models have better accuracy beyond 1000 copies/microliter for more than 20% variation. Later sections show the performance of the model with experimental data.

#### Measuring The Model Parameters For QuantStudio 3D Digital PCR Platform

Using an L20 pipette, 12.5 or 14.5 μL (nominal) reaction mix was loaded onto the chips and the chips sealed using the standard procedure. The actual volume loaded was determined with a gravimetric study on the actual volume of reaction mix dispensed by the pipette. The volume loaded that is used for the average well calculation is the adjusted sample volume, adjusted for the difference in weight. No liquid was left over after loading and no liquid in between the through-holes was observed, ensuring zero dead volume in these measurements. Five different lots of chips were used. The average well volume for each chip was determined by dividing the adjusted sample volume by the number of filled wells.

#### Measuring the Average Effective Load Volume

**Figure 3:**
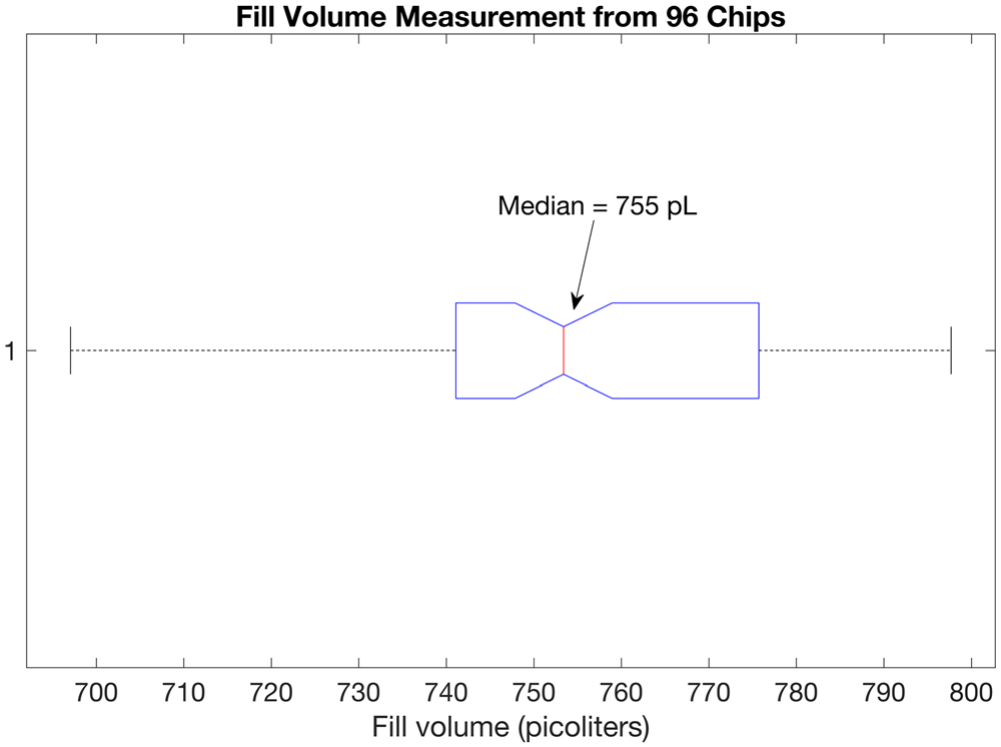
Median fill volume measured from 96 chips [Part #100027736].

Digital PCR experiments were conducted on the QuantStudio 3D Digital PCR System to measure the average through-hole fill volume. Experiments here present volume distribution profiles independent of instrument. Since the final quantification result is obtained by converting the number of molecules per partition to the number of molecules per unit volume, an accurate estimate of the mean volume has a large impact on the result. Corbisier *et al*.^7^ provide empirical evidence highlighting the importance of partition size in digital PCR quantification. Gravimetric measurements were made to accurately estimate the mean fill volume. The details of the materials and methods used in this experiment are included in Methods. The total volume loaded on the chip was divided by the total number of through-holes receiving master mix as indicated by their ROX fluorescence. Figure 3 shows the median fill volume measured from 96 chips. The blue box encloses the data between the 1^st^ and 3^rd^ quartile. The tails extend to 1.5 times the interquartile range. There are no outliers observed in this dataset. The median value of this dataset at 755 picoliters is chosen to represent the average through-hole fill volume. The mean fill volume at 754.72 picoliters, with a 3.03 percent coefficient of variation, was not much different from the median measurement. Note that even though array partition sizes are highly uniform, there may be additional factors that impact the fill volume that is accrued in each partition. It is this variation that is reflected in the measurements in Fig. 3 and we can correct for it with the modeling proposed in this paper.

#### Measuring the Coefficient of Variation for Effective Load Volume

For the QuantStudio 3D Digital PCR System, a direct measurement of the effective through-hole fill volume was challenging. A proxy measurement from every through-hole was used instead. Since the ROX dye is present in the master mix and quantified separately for each through-hole, the coefficient of variation in the ROX measurement could potentially be used as a proxy measure. Experiments here present a volume distribution profile independent of instrument.

**Figure 4:**
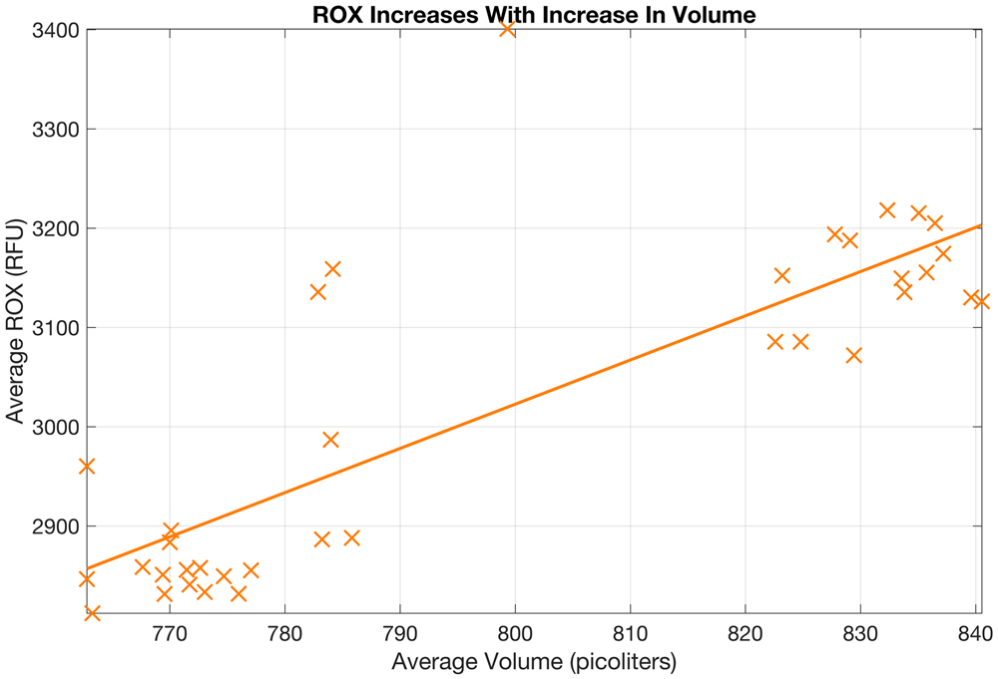
Relationship of average well fill volume to average ROX fluorescence from the chip center using 36 chips is presented. ROX increases with increase in volume with a correlation coefficient of 0.81. Hence, the variation in volume IS REFLECTED by variation in ROX. A linear relationship between average fill volume and average ROX is hypothesized. Specifically, in this dataset, the linear fit is given by: R = 4454.4*v − 541.

Figure 4 demonstrates the relationship between the average ROX fluorescence and the average fill volume using 36 chips. ROX fluorescence increases with increase in fill volume with a correlation coefficient of 0.81. As higher volumes have higher numbers of ROX fluorophores, it makes logical sense that higher volume shows higher ROX.

ROX measurements used to compute the average and the variance are taken from a single population of through-holes: either positives or negatives. If a negative population with more than 50 points is available in the central region, those are used. Otherwise, the population of positives in the central region is used. The edge areas of the chip are avoided because they are more susceptible to a variety of edge effects such as evaporation. The central part of the chip is more resistant to effects from evaporation. A set of chips are manufactured together on a wafer. It is seen that the average volume measured between adjacent chips manufactured on same wafer using all wells, do not show a significant variance. Thus, the estimates shown here based upon through-hole from the central region of the chip can be reliably applied to the whole chip.

A linear relationship between average volume and average ROX is hypothesized. Specifically, in this dataset, the linear fit is given by Eq. (14).

14 $${\rm{R}}=4454.4\times {\rm{v}}-541$$

Now, taking the mean and standard deviations, we get:

15 $$\bar{{\rm{R}}}=4454.4\times \bar{{\rm{v}}}-541$$

16 $${\rm{std}}({\rm{R}})=4454.4\times {\rm{std}}({\rm{v}})$$

Algebraic rearrangement yields the expression for the coefficient of variation as:

17 $${{\rm{CV}}}_{{\rm{R}}}=\frac{{\rm{std}}({\rm{R}})}{\bar{{\rm{R}}}}=(\frac{4454.4\times {\rm{std}}({\rm{v}})}{4454.4\times \bar{{\rm{v}}}-541})=(\frac{4454.4}{4454.4-\frac{541}{\overline{\bar{{\rm{v}}}}}})\times {{\rm{CV}}}_{{\rm{v}}}$$

Assuming a mean through-hole fill volume of 755 picoliters:

18 $${{\rm{CV}}}_{{\rm{R}}}=1.0002\times {{\rm{CV}}}_{{\rm{v}}}$$

It is thus reasonable to assume CV in ROX is representative of the CV in through-hole fill volume. In QuantStudio 3D AnalysisSuite Cloud Software, as ROX readings are available for each chip, the coefficient of variation is estimated on a per chip basis, which is, in turn, used to correct for the level of variation that is present for each chip analyzed. The mean volume used is fixed as part of the manufacturing specification, estimated as discussed in last section using gravimetric measurements.

Note that the chips here (Fig. 4) were deliberately overloaded and under loaded to tease out the relationship of the observable ROX measurement with the fill volume, to establish that ROX levels are acceptable as a proxy measure for through-hole fill volume. Therefore, the fill volume here is different from the 755 picoliters reported earlier (Fig. 3), where the intent of the experiment was to establish the mean fill volume when an experiment is performed following recommended protocol.

## Discussion

A set of samples was quantified using digital PCR on the QuantStudio 3D Digital PCR system. Details on the experimental material and methods are presented in Methods per recommended practices ^8^.

The positive and negative calls for each qualified through-hole were made using the QuantStudio 3D AnalysisSuite Cloud Software. The standard Poisson and the proposed Poisson Plus models were applied to get the quantification results.

**Figure 5:**
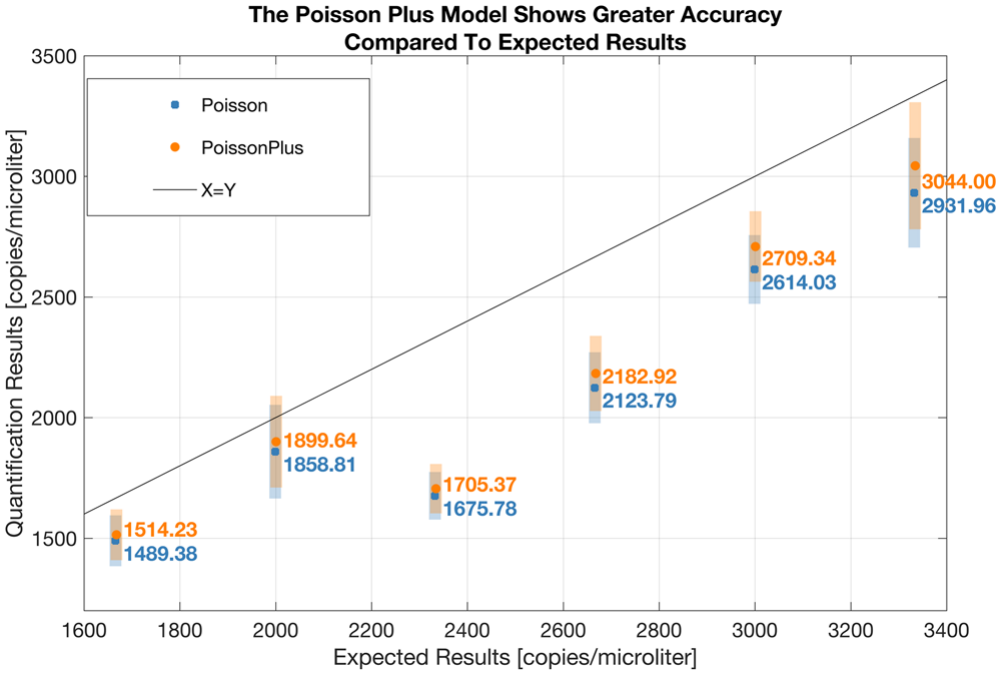
The p values from a t-test between results from the Poisson and the Poisson Plus modeling, at each concentration, is given as follows: 0.00000117, 0.04, 0.000003, 0.0002, 0.000004. The results suggest a significant difference in the means of the two distributions at the 95% confidence level at these concentrations. The mean result from each model along with a ± one standard deviation around this mean value is shown in the figure. The Poisson Plus results are consistently closer to the line of equivalence constructed with the expected results, suggesting a greater accuracy of the Poisson Plus Model at these concentrations.

Two sets of experiments are presented. Up to 6 replicate chips were run at each concentration shown in the first set of experiments. Figure 5 uses data from the first set of experiments. The p values from a t-test between results from the Poisson and the Poisson Plus modeling at each concentration is given as follows: 0.00000117, 0.04, 0.000003, 0.0002, 0.000004. The results suggest a significant difference in the means of the two distributions at the 95% confidence level at these concentrations. Two outliers were excluded at the third concentration because of which a p value could not be computed. Figure 5 suggests that the Poisson Plus modeling is more accurate than Standard Poisson as the Poisson Plus results lie closer to the line of equivalence (where the quantification perfectly matches the expected results).

**Figure 6:**
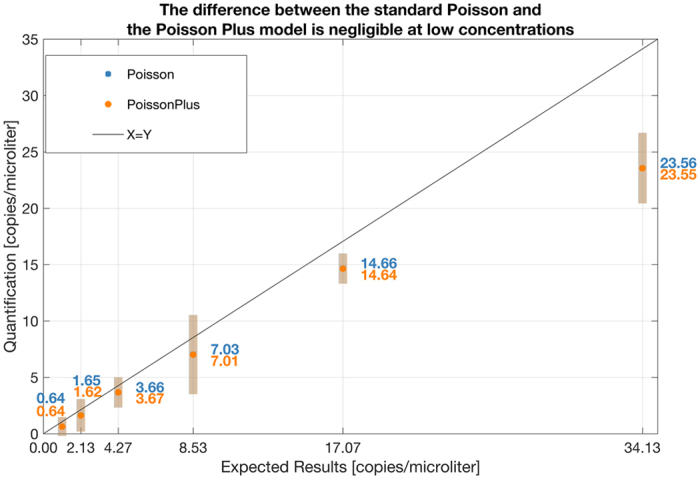
The p values from a t-test between results from the Poisson and the Poisson Plus modeling, at each concentration is given as follows: 0.8585, 0.4001, 0.7380, 0.0087, 0.5780, 0.9773. The results suggest that the difference between the standard Poisson and the Poisson Plus Model are insignificant at the 95% confidence level at these low concentrations. The mean result from each model along with the ± one standard deviation around this mean value is shown in the figure.

Figure 6, constructed from the same set of experiments as Fig. 5, focusses on a different concentration range. The p values from a t-test between results from the Poisson and the Poisson Plus modeling at each concentration is given as follows: 0.8585, 0.4001, 0.7380, 0.0087, 0.5780, 0.9773. The results suggest that the difference between the standard Poisson and the Poisson Plus Model are insignificant at the 95% confidence level at these low concentrations.

**Figure 7:**
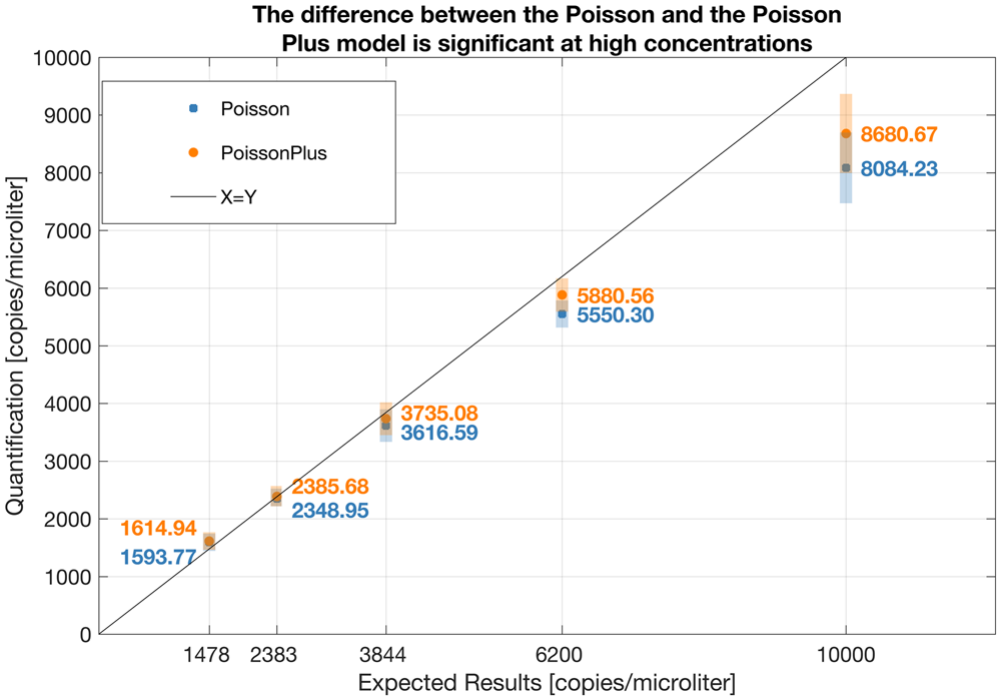
The p values from a t-test between results from the Poisson and the Poisson Plus modeling at each concentration is given as follows: 0.00000117, 0.04, 0.000003, 0.0002, 0.000004. The results suggest a significant difference in the mean results produced by Poisson and Poisson Plus modeling at the 95% confidence level at these concentrations. The mean result from each model along with a ± one standard deviation around this mean value is shown in the figure. The majority of the mean Poisson Plus results are closer to the line of equivalence constructed with the expected results, suggesting a greater accuracy of the Poisson Plus Model at these concentrations.

Figure 7, constructed from the second set of experiments show that the standard Poisson and the Poisson Plus Model produce a much higher difference in results at the higher concentration. The p values from a t-test between results from the Poisson and the Poisson Plus modeling at each concentration is given as follows: 0.00000117, 0.04, 0.000003, 0.0002, 0.000004. The results suggest a significant difference in the mean results produced by Poisson and Poisson Plus modeling at the 95% confidence level at these concentrations. The majority of the mean Poisson Plus results are closer to the line of equivalence constructed with the expected results, suggesting a greater accuracy of the Poisson Plus Model at these concentrations.

Note that there are three effects. The standard Poisson model underestimates by larger amounts as the concentration increases; therefore, the magnitude of the correction from models taking load variation into account also increases with concentration. The second effect is that, a given chip has a certain load variation. The larger that load variation, the larger is the magnitude of the correction. This is independent of the concentration. The spread of the data for each concentration in the figure is a third effect, which reflects the normal experimental variation, independent of both the concentration and the extent of volume variation measured on the chip.

**Figure 8:**
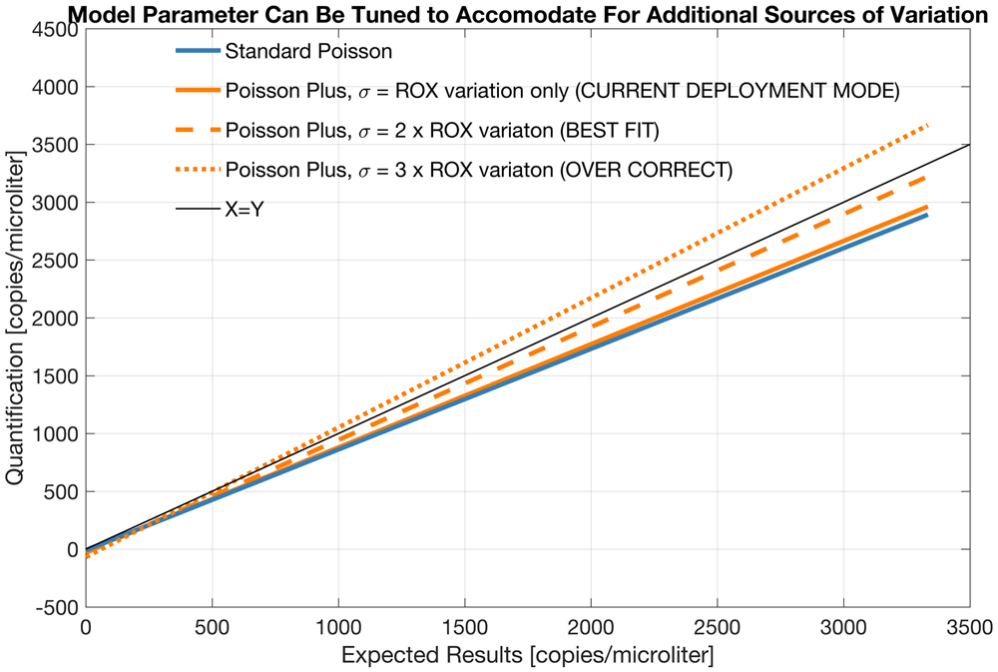
Recomputed results using a fixed scaling factor applied to the ROX variation assessed from each chip. When the sigma parameter in Poisson Plus is set to twice the coefficient of variation in ROX, assessed on a per chip basis, the linear fit of the data lies almost on top of the X = Y line, demonstrating the efficacy of this correction when the levels of variation is appropriately characterized.

Note that sources of variation other than volumetric variation, can, in principle be all lumped into the sigma parameter of the Poisson Plus model. Figure 8 shows the recomputed results using a fixed scaling factor applied to the ROX variation assessed from each chip. When the sigma parameter in Poisson Plus is set to twice the coefficient of variation in ROX, assessed on a per chip basis, the linear fit of the data lies almost on top of the X = Y line, demonstrating the efficacy of this correction when the levels of variation is appropriately characterized. However, this is not a strategy we adopted in the software, as the characterization of the appropriate factor would require much larger studies, not available to us at this time.

Poisson based quantification is sensitive to effective load variability, particularly at higher concentrations. A novel modeling for estimating the concentration is presented in this paper for the case where there is finite variation in effective load volume of each partition. We have shown that models taking this variation into account can be harnessed to reproducibly quantify results despite the variation. The key to the success of these models is in the correct assessment of the true levels of variation of the effective load volume. Data demonstrate improved precision and comparable linearity at the higher end of the concentration spectrum between the uses of the Poisson Plus verses the Poisson models for quantification.

While the described Poisson Plus models may fail if the partitions aren’t distributed following a truncated normal or normal distribution, the general approach to addressing the problem will remain valid. However, new expressions for the concentration will need to be derived using a suitable partition size distribution model.

The QuantStudio 3D Digital PCR System and the QuantStudio 3D AnalysisSuite Cloud Software from Thermo Fisher Scientific are For Research Use Only. Not for use in diagnostic procedures.

## Methods

### Experiment I

Data in Figs 3 and 4.

#### Assays

TaqMan® Copy Number Reference Assay, RNase P, Human (VIC), (PN4403326); Hs04107718_cn, TaqMan® Copy Number Assay, GLA (Thermo Fisher Scientific).

#### Sample

Control DNA (from CEPH Individual 1347-02), (PN 403062) (Thermo Fisher Scientific), diluted to approximately 100 copies/μL RNaseP and 200 copies/μL of GLA.

#### Protocol

Reaction mixtures were prepared by combining genomic DNA, RNase P assay, GLA assay, and QuantStudio™ 3D Digital PCR Master Mix v2 (PN A26358), according to the manufacturer’s instructions. Each reaction mixture was loaded onto a QuantStudio 3D Digital PCR Chip v2 (PN 100027736).

For all experiments, chips were run on a ProFlex™ 2 x flat PCR System (Applied Biosystems™) cycled with the following conditions: 10 min at 96.0 degree C; then 39 cycles at 60.0 degree C for 2 min, 30 s at 98.0 degree C; and a final elongation step of 2 min at 60 degree C. End-point fluorescence data were collected on the QuantStudio 3D Digital PCR instrument and analyzed using the QuantStudio 3D AnalysisSuite software (Thermo Fisher Scientific).

### Experiment II

Data in Figs 5 and 6.

#### Assays

BCR-ABL1 primers and probe: ENF501_F (5′-TCCGCTGACCATCAAYAAGGA-3′), ENF561_R (5′-CACTCAGACCCTGAGGCTCAA-3′), ENP541 (6FAM-5′-CCCTTCAGCGGCCAGT-3′-MGB).

ABL1 primers and probe: ENF1003_F (5′-TGGAGATAACACTCTAAGCATAACTAAAGGT-3′), ABL1063_R (5′-GATGTAGTTGCTTGGGACCCA-3′), ABL1043 (VIC-5′-CATTTTTGGTTTGGGCTTC-3′-MGB).

#### Sample

A dilution series was prepared using the ERM-AD623 plasmid (produced by the European Commission for Reference Materials), a reference vector containing the BCR-ABL1 exonic fusion and ABL1 seqeunce.

#### Protocol

Reaction mixtures were prepared by combining plasmid, BCR-ABL1 assay and ABL1 assay at a final primer concentration of 900 nM each and probes at a final concentration of 250 nM, and QuantStudio™ 3D Digital PCR Master Mix (PN 4482710), according to the manufacturer’s instructions. Each reaction mixture was loaded onto a QuantStudio 3D Digital PCR Chip (PN 4482954) for quantification.

### Experiment III

Data in Fig. 7.

#### Assays

TaqMan® Copy Number Reference Assay, RNase P, Human (VIC), Catalog # 4403326; (Thermo Fisher Scientific).

#### Sample

Varying amounts of a plasmid containing the amplicon of the RNase P assay.

#### Protocol

Reaction mixtures were prepared by combining dilution series of plasmid DNA, RNase P assay, and QuantStudio™ 3D Digital PCR Master Mix v2 (Cat # A26358), according to the manufacturer’s instructions. Each reaction mixture was loaded onto a QuantStudio 3D Digital PCR Chip v2 (PN 100027736) for quantification.

## Conclusion

The Poisson-Plus Model for quantification of results of a digital PCR experiment, first released to public in July 2015 as part of the QuantStudio 3D AnalysisSuite Cloud Software from Thermo Fisher Scientific, is presented. The model accommodates for the underestimation caused by using standard Poisson model in the presence of fill volume variation of individual reactions. The fill volume variation was measured on the chip array based QuantStudio 3D Digital PCR System using the ROX fluorescence level as a proxy for effective load volume per through-hole. Monte Carlo simulations demonstrated the efficacy of the proposed correction. The model was used to analyze digital PCR experiments and showed improved accuracy in quantification. Particularly at the higher concentrations, the modeling takes effective fill volume variation into account to produce more accurate estimates. The extent of the difference from the standard to the new modeling is positively correlated to the extent of fill volume variation in the effective load of the reactions.

## Electronic supplementary material

Appendix
