# Supplementary material for: Poisson Plus Quantification for Digital PCR Systems
Source: Sci Rep. 2017 Aug 29;7:9617. doi: 10.1038/s41598-017-09183-4 (PMC5575031; doi:10.1038/s41598-017-09183-4)
Supplement: Supplementary file 1 — Appendix [file 41598_2017_9183_MOESM1_ESM.pdf]

Poisson Plus Quantification for Digital PCR Systems

Nivedita Majumdar[1,*], Swapnonil Banerjee, Michael Pallas[1], Thomas Wessel[1], Patricia Hegerich[1]

[1]ThermoFisher Scientific, Life Sciences Group, South San Francisco, 94080, USA
*nivedita.majumdar@thermofisher.com

Appendix A

Derivation of $P(neg)$ in (6)

$$\mathrm{P(neg)} = \int_{-\infty}^{\infty} \mathrm{P\,(neg, v)dv}$$

$$= \frac{1}{\sigma\sqrt{2\pi}} \int_{-\infty}^{\infty} e^{-Cv} e^{-\frac{-(v-v_0)^2}{2\sigma^2}} dv$$

$$= \frac{1}{\sigma\sqrt{2\pi}} \int_{-\infty}^{\infty} e^{-Cv} e^{-\frac{[v^2-2vv_0+v_0^2]}{2\sigma^2}} dv$$

$$= \frac{1}{\sigma\sqrt{2\pi}} e^{-\frac{v_0^2}{2\sigma^2}} \int_{-\infty}^{\infty} e^{-Cv} e^{-\frac{v^2}{2\sigma^2}} e^{\frac{vv_0}{\sigma^2}} dv$$

$$= \frac{1}{\sigma\sqrt{2\pi}} e^{-\frac{v_0^2}{2\sigma^2}} \int_{-\infty}^{\infty} e^{-\frac{v^2}{2\sigma^2}} e^{v(\frac{v_0}{\sigma^2}-C)} dv \qquad \text{(i)}$$

It is known that from Feynman, et al [7] that:

$$\int_{-\infty}^{\infty} e^{ax^2+bx} dx = \sqrt{\pi/_{-a}}\, e^{-b^2/_{4a}} \qquad \text{(ii)}$$

Using $a = -\frac{1}{2\sigma^2}$ and $b = \frac{v_0}{\sigma^2} - C$ in (ii), (i) becomes:

$$\mathrm{P(neg)} = \frac{1}{\sigma\sqrt{2\pi}} e^{-\frac{v_0^2}{2\sigma^2}} \sqrt{\pi(2\sigma^2)}\, e^{\frac{-\left(\frac{v_0}{\sigma^2}-C\right)^2}{4\left(-\frac{1}{2\sigma^2}\right)}}$$

$$= \frac{1}{\sigma\sqrt{2\pi}} \frac{\sqrt{\pi(2\sigma^2)}\, e^{\left(\frac{v_0^2}{\sigma^4}+C^2-\frac{2Cv_0}{\sigma^2}\right)\frac{\sigma^2}{2}}}{e^{\frac{v_0^2}{2\sigma^2}}}$$

$$= \frac{1}{\cancel{\sigma\sqrt{2\pi}}} \frac{\cancel{\sqrt{\pi(2\sigma^2)}}\, e^{\left(\frac{v_0^2}{\sigma^4}+C^2-\frac{2Cv_0}{\sigma^2}\right)\frac{\sigma^2}{2}}}{e^{\frac{v_0^2}{2\sigma^2}}}$$

$$= \frac{\cancel{e^{\frac{v_0^2}{2\sigma^2}}}\, e^{\left(\frac{C^2\sigma^2}{2}-\frac{\cancel{2}Cv_0\cancel{\sigma^2}}{\cancel{\sigma^2}\ \cancel{2}}\right)}}{\cancel{e^{\frac{v_0^2}{2\sigma^2}}}}$$

$$= e^{(\frac{1}{2}\sigma^2C^2-Cv_0)}$$ (iii), which is the same as (6).

Derivation of C, as given by (7)

The concentration *C* can be solved from (6) as follows:

$$\mathrm{P(neg)} = \exp\left(\frac{1}{2}\sigma^2C^2 - Cv_0\right) \qquad \text{(v)}$$

Taking logarithm on both sides of (v) and rearranging we obtain:

$$\frac{1}{2}\sigma^2 C^2 - Cv_0 - \ln P(neg) = 0 \qquad \text{(vi)}$$

Solving for C from the above equation, we obtain

$$C = \frac{v_0 \pm \sqrt{v_0^2 + 2\sigma^2 \ln P(neg)}}{\sigma^2}$$

(vii), which is the same as (7).

Derivation of (8) from (7) keeping the negative sign only

C in (7) can be written as:

$$= \frac{v_0 \pm v_0 \sqrt{1 + \frac{2\sigma^2}{v_0^2}\ln(P_{neg})}}{\sigma^2} \qquad \text{(viii)}$$

Using the fact that $(1+x)^{1/2} \approx 1 + \frac{x}{2}$ for small x, C can be simplified as:

$$C \approx \frac{v_0 \pm v_0 (1 + \frac{\sigma^2}{v_0^2}\ln(P_{neg}))}{\sigma^2} \qquad \text{(ix)}$$

Using only the negative sign in (ix), we obtain

$$C \approx \frac{v_0 - v_0 - \frac{\sigma^2}{v_0}\ln(P_{neg})}{\sigma^2} = \frac{-\ln(P_{neg})}{v_0}$$

(x), which is same as (8).

Appendix B Derivation of (13)

$$P(neg) = \int_0^\infty P(neg, v)dv \qquad \text{(i)}$$

Using $P(neg, v)$, as given by (11), in (i), we obtain

$$P(neg) = \frac{1}{\sigma\sqrt{\frac{\pi}{2}}\operatorname{erfc}\left[-\frac{1}{\sqrt{2}}\left(\frac{v_0}{\sigma}\right)\right]} \int_0^\infty e^{-Cv} e^{-\frac{(v-v_0)^2}{2\sigma^2}} dv$$

$$= \frac{1}{\sigma\sqrt{\frac{\pi}{2}}\operatorname{erfc}\left[-\frac{1}{\sqrt{2}}\left(\frac{v_0}{\sigma}\right)\right]} I \qquad \text{(ii)}$$

where

$$I = \int_0^\infty e^{-Cv} e^{-\frac{(v-v_0)^2}{2\sigma^2}} dv.$$

$$= \int_0^\infty e^{-Cv} e^{-\frac{(v^2 - 2vv_0 + v_0^2)}{2\sigma^2}} dv$$

$$= e^{-\frac{v_0^2}{2\sigma^2}} \int_0^\infty e^{-Cv} e^{-\frac{(v^2 - 2vv_0)}{2\sigma^2}} dv$$

$$= e^{-\frac{v_0^2}{2\sigma^2}} \int_0^\infty e^{-\frac{v^2}{2\sigma^2}} e^{v\left(\frac{v_0}{\sigma^2} - C\right)} dv$$

$$= e^{-\frac{v_0^2}{2\sigma^2}} \int_0^\infty e^{-\mathcal{H}v^2 + \mathcal{M}v} dv \qquad \text{(iii)}$$

where

$$\mathcal{H} = \frac{1}{2\sigma^2}, \mathcal{M} = \frac{v_0}{\sigma^2} - C. \qquad \text{(iv)}$$

Let

$$I' = \int_0^\infty e^{-\mathcal{H}v^2 + \mathcal{M}v} dv$$

$$= \int_0^\infty e^{-\mathcal{H}\left(v^2-\frac{\mathcal{M}}{\mathcal{H}}v\right)}dv$$

$$= \int_0^\infty e^{-\mathcal{H}\left(v^2-2v\frac{\mathcal{M}}{2\mathcal{H}}+\frac{\mathcal{M}^2}{4\mathcal{H}^2}-\frac{\mathcal{M}^2}{4\mathcal{H}^2}\right)}dv$$

$$= \int_0^\infty e^{-\mathcal{H}\left(v-\frac{\mathcal{M}}{2\mathcal{H}}\right)^2} e^{\frac{\mathcal{M}^2}{4\mathcal{H}}}dv$$

Substituting $\mathcal{H}$ and $\mathcal{M}$ given by (iv)

$$= \int_0^\infty e^{-\frac{1}{2\sigma^2}\left(v-\frac{\frac{v_0}{\sigma^2}-C}{\frac{1}{\sigma^2}}\right)^2} e^{\frac{\left(\frac{v_0}{\sigma^2}-C\right)^2}{4\frac{1}{2\sigma^2}}}dv$$

$$= e^{\frac{\sigma^2}{2}\left(\frac{v_0}{\sigma^2}-C\right)^2}\int_0^\infty e^{-\frac{1}{2\sigma^2}\left(v-\sigma^2\left(\frac{v_0}{\sigma^2}-C\right)\right)^2}dv$$

$$= e^{\frac{\sigma^2}{2}\left(\frac{v_0{}^2}{\sigma^4}-2C\frac{v_0}{\sigma^2}+C^2\right)}\int_0^\infty e^{-\frac{1}{2\sigma^2}\left(v-v_0+C\sigma^2\right)^2}dv$$

$$= e^{\frac{v_0{}^2}{2\sigma^2}}e^{-Cv_0+\frac{1}{2}\left(\sigma^2C^2\right)}\int_0^\infty e^{-\frac{1}{2\sigma^2}\left(v-v_0+C\sigma^2\right)^2}dv \quad \text{(v)}$$

Using (v) in (iii), we obtain:

$$I = e^{-Cv_0+\frac{1}{2}\left(\sigma^2C^2\right)}\int_0^\infty e^{-\frac{1}{2\sigma^2}\left(v-v_0+C\sigma^2\right)^2}dv \quad \text{(vi)}$$

Let

$$I'' = \int_0^\infty e^{-\frac{1}{2\sigma^2}\left(v-v_0+C\sigma^2\right)^2}dv \quad \text{(vii)}$$

Making the substitution of variable

$$v'' = \frac{1}{\sqrt{2}\sigma}\left(v-\left(v_0-C\sigma^2\right)\right) \quad \text{(viii)}$$

in (vii), we obtain,

$$I'' = \int_{-\frac{(v_0-C\sigma^2)}{\sqrt{2}\sigma}}^\infty e^{-v''^2}\sqrt{2}\sigma dv''$$

$$= \sqrt{2}\sigma\frac{\sqrt{\pi}}{2}\frac{2}{\sqrt{\pi}}\int_{-\frac{(v_0-C\sigma^2)}{\sqrt{2}\sigma}}^\infty e^{-v''^2}dv''$$

$$= \sqrt{2}\sigma\frac{\sqrt{\pi}}{2}\operatorname{erfc}\left(-\frac{(v_0-C\sigma^2)}{\sqrt{2}\sigma}\right) \quad \text{(ix)}$$

Using (vi) and (ix) in (ii), we obtain

$$P(\text{neg})$$

$$= \frac{1}{\sigma\sqrt{\frac{\pi}{2}}\operatorname{erfc}\left[-\frac{1}{\sqrt{2}}\left(\frac{v_0}{\sigma}\right)\right]}\sqrt{2}\sigma\frac{\sqrt{\pi}}{2}\operatorname{erfc}\left[-\frac{1}{\sqrt{2}}\left(\frac{v_0}{\sigma}-C\sigma\right)\right]e^{-Cv_0+\frac{1}{2}\sigma^2C^2}$$

$$= \frac{\operatorname{erfc}\left[-\frac{1}{\sqrt{2}}\left(\frac{v_0}{\sigma}-C\sigma\right)\right]}{\operatorname{erfc}\left[-\frac{1}{\sqrt{2}}\left(\frac{v_0}{\sigma}\right)\right]}e^{-Cv_0+\frac{1}{2}\sigma^2C^2} \quad \text{(x)}$$

which is the same as (13).

Appendix C Steps of the simulations

1) Generate a normal distribution with a given mean and coefficient of variation

↓

2) For each concentration, find probability p, of 0 molecules/partition, assuming uniform partition sizes. The probability of a partition to obtain a molecule is made proportional to the size of the partition. So larger partitions have the greater chance of containing a molecule.

↓

3) Generate a random number r between 0 and 1. If $r < p$, then no molecules are assumed to be in that partition.

↓

4) Quantify the number of molecules per partition from this population of partitions with at least one or no molecules. Repeat over the number of iterations.
